# Discovery of Fibrinogen γ-chain as a potential urinary biomarker for renal interstitial fibrosis in IgA nephropathy

**DOI:** 10.1186/s12882-023-03103-7

**Published:** 2023-03-20

**Authors:** Jie Guan, Meiling Wang, Man Zhao, Wentao Ni, Man Zhang

**Affiliations:** 1 Peking University Ninth School of Clinical Medicine, Beijing, China; 2 Clinical Laboratory Medicine, Beijing Shijitan Hospital, Capital Medical University, No. 10 Tieyi Road, Haidian District, Beijing, 100038 China; 3 Beijing Key Laboratory of Urinary Cellular Molecular Diagnostics, Beijing, China; 4 Department of Clinical Laboratory, Peking University First Hospital, Xicheng District, Beijing, China; 5 Department of Pulmonary and Critical Care Medicine, Peking University People’s Hospital, Xicheng District, Beijing, China

**Keywords:** Urinary biomarker, IgA nephropathy, Renal interstitial fibrosis, Fibrinogen γ-Chain, Data independent acquisition

## Abstract

**Background:**

IgA nephropathy (IgAN) is a major cause of chronic kidney disease (CKD). Renal interstitial fibrosis is a hallmark of CKD progression. Non-invasive biomarkers are needed to dynamically evaluate renal fibrosis. Data independent acquisition (DIA)-based liquid chromatography-mass spectrometry (DIA-MS) was used to identify candidate urinary biomarkers in IgAN patients with different renal interstitial fibrosis degrees.

**Methods:**

Eighteen biopsy-proven IgAN patients and six healthy controls were recruited in a discovery cohort. Interstitial fibrosis changes were evaluated according to Oxford MEST-C scores. Urinary samples were analyzed with DIA-MS to identify hub proteins. Hub proteins were then confirmed by enzyme-linked immunosorbent assay (ELISA) in a validation cohort and the associated gene mRNA expression was analyzed using public gene expression omnibus (GEO) datasets.

**Results:**

Complement and coagulation cascades pathway was the main KEGG pathway related to the over-expressed proteins. Fibrinogen γ-Chain (FGG) was selected as the potential urinary marker for further validation. Urinary FGG to creatinine ratio (uFGG/Cr) levels were higher in both disease controls and IgAN group than in healthy controls, but were not significantly different between IgAN and disease groups. uFGG/Cr was confirmed to be increased with the extent of renal fibrosis and presented moderate correlations with T score (r = 0.614, p < 0.01) and eGFR (r = -0.682, p < 0.01), and a mild correlation with UTP (r = 0.497, p < 0.01) in IgAN group. In disease control group, uFGG/Cr was higher in patients with T1 + 2 compared to those with T0. uFGG/Cr had a good discriminatory power to distinguish different fibrosis stages in IgAN: interstitial fibrosis ≤ 5% (minimal fibrosis) vs. interstitial fibrosis (mild fibrosis) > 5%, AUC 0.743; T0 vs. T1 + 2, AUC 0.839; T0 + 1 vs. T2, AUC 0.854. In disease control group, uFGG/Cr showed better performance of AUC than UTP between minimal and mild fibrosis (p = 0.038 for Delong’s test). Moreover, GSE104954 dataset showed that FGG mRNA expression was up-regulated (fold change 1.20, p = 0.009) in tubulointerstitium of IgAN patients when compared to healthy living kidney donors.

**Conclusion:**

Urinary FGG is associated with renal interstitial fibrosis and could be used as a noninvasive biomarker for renal fibrosis in IgAN.

**Supplementary Information:**

The online version contains supplementary material available at 10.1186/s12882-023-03103-7.

## Introduction

Immunoglobulin A nephropathy (IgAN) is a primary cause of chronic kidney disease (CKD) worldwide. In China, IgAN accounts for nearly 50% of primary glomerulonephritis, and is a major factor leading to end-stage renal disease (ESRD) [1]. The histological manifestation of IgAN is characterized as predominant deposition of IgA in glomerular mesangial region with mesangial cell proliferation and mesangial matrix expansion [2, 3]. Although the mechanism has not been elucidated, it is demonstrated that a decline in renal function and IgAN progression are associated with renal interstitial fibrosis [4–7].

Interstitial fibrosis is considered as a common hallmark of progressive kidney disease with no disease specificity. After kidney injury, protein secretion, degradation, and synthesis in extracellular matrix (ECM) are unbalanced, leading to unchecked fibrotic deposition, which amplifies the severity of kidney injury [8]. In the process of renal fibrosis, multiple protein expression alterations are related to the subsequent pathomorphological changes [9]. Proteins are the direct effectors of both biological processes and almost all drug targets, thus proteomics techniques could be used to identify potential biomarkers for the evaluation of chronic kidney disease or renal interstitial fibrosis [10, 11]. However, renal biopsy is an invasive technique and it is difficult to be accepted by all patients especially for monitoring purpose. So urinary biomarkers have a great potential as urine is an important specimen source for renal diseases. Numerous studies have identified individual or combined urinary biomarkers for the evaluation of renal interstitial fibrosis with proteomics technique [12–15]. Urinary epidermal growth factor (EGF) was identified by Liquid Chromatography with tandem mass spectrometry (LC-MS/MS) to be associated with renal interstitial fibrosis and long-term adverse kidney outcomes in lupus nephritis [12]. A panel of 273 urinary peptide biomarkers of CKD (CKD273) was developed by capillary electrophoresis coupled to mass spectrometry (CE-MS) [16]. It was demonstrated to be correlated with tubulo-interstitial fibrosis in a lupus nephritis cohort and was of great prognostic value in a large multicenter CKD cohort [15, 17]. CKD273 and a recently developed 29-peptide classifier were also identified to be correlated with renal fibrosis degrees in cohorts composed of various types of CKD patients including IgAN [13, 14].

Clinical-pathological patterns are heterogeneous in IgAN and a wide spectrum of clinical and pathologic features have been reported [4]. Yet there is a lack of urine markers for the early evaluation of interstitial fibrosis in IgAN. In this study we aimed to identify specific urinary biomarkers related to renal fibrosis in IgAN by using data-independent acquisition based liquid chromatography-mass spectrometry (DIA-MS) (Additional file 1).

## Materials and methods

### Study design

This cross-sectional observational study was performed from May 2021 to November 2021. All subjects in this study were divided into discovery cohort and validation cohort and all patients met the following inclusion criteria: (i) adult patients (> 18-year-old); (ii) diagnosis of IgAN confirmed by renal biopsy (Oxford classification); (iii) diagnosis of other CKD patients confirmed by renal biopsy; (iv) no family history of kidney disease; (v) no medical history of liver, lung or heart diseases. The study was conducted in compliance with the declaration of Helsinki principles and followed the recommendations of Medical Ethics Committee of Peking University First Hospital and all subjects provided written consent before inclusion (2021KEYAN002). Healthy controls (HC) met the following inclusion criteria: (i) > 18 years old; (ii) apparently healthy individuals; (iii) no medical history of kidney, liver, lung or heart diseases.

### Data collection

Clinical and laboratory data were measured and collected in Clinical Laboratory of Peking University First Hospital. Serum creatinine, estimated glomerular filtration rate (eGFR), plasma hemoglobin (Hgb), serum triglyceride, total cholesterol and 24-hour urinary total protein were the main clinical characteristics and were measured one day before kidney biopsy. eGFR was calculated according to Chronic Kidney Disease Epidemiology Collaboration equation [18].

### Evaluation of interstitial fibrosis

Oxford classification MEST-C system was used to evaluate the interstitial fibrosis for IgAN patients. Other CKD patients in disease control group were only evaluated with T scores. T-score in MEST-C was defined as: T0 ≤ 25%, T1: 26–50%, T2: > 50%. In this study, we further divided T0 into minimal ≤ 5% and mild 6-25% according to Banff classification [19].

### Urine sample collection and preparation

Random urine samples were collected on the same day of renal biopsy and centrifuged (400×g, 5min) to remove cellular debris within 4 hours of collection. Urine supernatants were stored at -80℃ for further analysis. Before proteomics analysis, urine samples in discovery cohort were thawed and pooled urine samples in each group were obtained by mixing 600µl urine sample of each subject. Then the mixed urine samples in each group were divided into three aliquots for subsequent proteomics analysis.

### Urinary protein extraction and tryptic digestion

An equal volume of each urine sample was diluted with lysis buffer (50Mm NH4HCO_3_, pH7.4, 10 Mm MgCl_2_, 7M urea, 2M thiourea), followed by a 10KD ultrafiltration (Sartorious, Göttingen, Germany) and centrifugation (12000 × g, 15min, 4℃). The supernatant protein was collected in new tubes and reduced with 20mM Dithiothreitol (DTT) for 30 min at 55℃ and alkylated with 40Mm iodoacetamide at 25 ℃ for 15 min. The protein was then digested with trypsin (Promega, Madison, USA) at 1:50 enzyme-to-protein ratio for 16 h at 37℃. The peptides were desalted using C18 tips and then dried by vacuum evaporation. Protein concentration was measured with Bradford assay. Pooled peptide sample was obtained by mixing equal number of digested peptides of each group sample for generating a spectral library.

### Offline High-pH reversed-phased peptide fractionation

The pooled peptide sample was redissolved with Buffer A (2% Acetonitrile, 98% H_2_O, pH 10.0) and fractionated using the L-3000 HPLC (RIGOL, Beijing, China) coupled with a Waters XBridge BEH C18 column (4.6 × 250mm, 5 µm). Peptides were separated with Buffer A and Buffer B (98% Acetonitrile, 2% H_2_O, pH 10.0) and fractions were collected per minute. The gradient was set as follows: 0–20 min, 5% B; 20–120 min, 5% B; 120–125 min, 40% B; 125–135 min, 95% B; 135–140 min, 95% B; 140–150 min, 5% B. The eluates were collected for a tube per minute and merged into 24 fractions. All fractions were dried by vacuum evaporation.

### DIA Proteomics Analysis

L-3000 HPLC system combined with Q Exactive HFX mass spectrometer (Thermo Fisher, Waltham, USA) and NanosprayFlex™ electrospray ionization (ESI) ion source was used for library generation in DDA mode. 24 dried fractions were resuspended respectively by 0.1% formic acid (FA) with and iRT standard peptide (Biognosys, Schlieren, Switzerland). In DDA mode, the full scan range was M/Z 350–1500, with resolution at 120,000, autogain control (AGC) at 3e6 and maximum injection time of 80 ms. MS/MS spectra were acquired at a resolution of 15000, with an AGC at 5e4, maximum injection time of 45 ms and a normalized collision energy of 27.

Samples of each group were resuspended respectively by 0.1% FA with iRT standard peptide (Biognosys, Schlieren, Switzerland). In DIA mode, the full scan range was m/z 350–1250, with resolution at 120,000, AGC at 3e6 and maximum injection time of 60 ms. MS/MS spectra were acquired at a resolution of 12000, with AGC at 3e6, maximum injection time of 60 ms and a normalized collision energy of 27, with 60 variable windows ranging from 349.5 to 1250.5 m/z.

### Proteomics Data Analysis

DDA raw data of 24 fractions were processed with Spectronaut^™^ 15 (Biognosys, Schlieren, Switzerland) to generate the spectra library. The false discovery rate (FDR) of protein identifications was set to 1.0%. All DIA files of urine samples were analyzed using Spectronaut with default settings and the library generated from the DDA runs in the same experiment. The proteomic data were not normalized in DIA analysis.

### Validation by ELISA analysis

Commercially available ELISA kits for fibrinogen γ-chain (FGG) (Cloud-Clone Corp, Houston, USA) and complement C9 (C9) (Fine Biotech Co., Ltd. Wuhan, China) were performed for quantitative analysis according to the manufacture’s protocols. Briefly, urine samples were thawed and diluted with PBS at a dilution of 1:20 for FGG and 1:10 for C9, then applied to individual wells and incubated on an incubator for 60 minutes. Wells were then incubated with anti-human FGG or C9 antibody for 60 minutes, followed by the incubation of anti-human IgG antibody for 30 minutes. The colorimetric detection was performed using a TMB/peroxide substrate incubation and the OD (450nm) at 15 minutes was read on a Multiskan MK3 (Thermo fisher, Waltham, USA). Finally, urinary FGG (uFGG) and C9 (uC9) concentrations were calculated based on simultaneous standard curves generated from standard materials. Urinary creatinine (uCr) was determined on AU5800 (Beckman, Brea, USA) and uFGG or uC9 were normalized with urinary creatinine.

### Transcriptomics data analysis

Microarray dataset GSE104954 was downloaded from the Gene Expression Omnibus (GEO) database (https://www.ncbi.nlm.nih.gov/geo/) using GEO query package in R (version 4.0.3) for transcriptional validation in tubulointerstitium from kidney biopsy of IgAN. This dataset consists of 25 IgAN patients and 21 living donors. Hub protein mRNA expression data were extracted and then normalized with the limma R package (version 3.46.0) for further analysis.

### Bioinformatics analysis

FDR < 0.05 (-Log_10_FDR > 1.3013) coupled with fold change (FC) > 1.5 or < 0.667 was set as a cutoff for filtering significantly expressed proteins. Heatmap was performed with ClustVis, a web tool for visualizing clustering of multivariate data (http://biit.cs.ut.ee/clustvis) [20]. Common differentially expressed proteins (DEPs) among multiple groups were identified with DrawVenndiagram (http://bioinformatics.psb.ugent.be/webtools/Venn/). ClusterProfiler version 4.4.1 package was applied to perform gene ontology (GO) function and Kyoto Encyclopedia of Genes and Genomes (KEGG) annotations [21, 22]. Protein‑protein interaction (PPI) networks were performed with the DAVID online database (https://david.ncifcrf.gov/). Then, the PPI was visualized in Cytoscape software (version: 3.8.1). Nonparametric Mann-Whitney test was used to analyze the difference of protein expression abundance in each group.

### Statistical analysis

Baseline parameters of participants were reported as median and interquartile range (IQR) due to the small number of subjects, and categorical variables were expressed by frequency (or percentage). Nonparametric Mann-Whitney U test or Kruskal-Wallis one way analysis of variance was used for differences of numerical data including baseline parameters, proteomic results and mRNA expression data among different groups using GraphPad Prism software (version 9.0.2). When Kruskal-Wallis one way analysis was performed, Dunn’s multiple comparisons test was used for post-hoc analysis. Spearman’s correlation analysis was performed among different variables in IgAN group with SPSS software (version 19.0). Receiver operating characteristics curves (ROC) was determined by SPSS software, with the area under the curve (AUC) compared using the paired DeLong’s test by MedCalc software (version 18.2.1).

## Results

### Patients

One hundred and thirty-six subjects were enrolled in this study. The discovery cohort consisted of sex-matched 18 IgAN patients with T0 to T2 and 6 healthy controls (Additional file 2). Another 50 IgAN patients, 42 other CKD patients and 20 healthy controls were enrolled in validation cohort. A summary of patient characteristics including histological type and laboratory values are shown in Table 1. There were 7 minimal change disease (MCD), 12 diabetic nephropathy (DN) and 23 membranous nephropathy (MN) patients in disease control (DC) group.

**Table 1:** Clinical characteristics and baseline biopsy evaluation in discovery and validation set

Variable	Discovery cohort					Validation cohort				
	IgAN group (n = 18)	Healthy control (n = 6)	P value		IgAN group (n = 50)		Disease controls (n = 42)	Healthy controls (n = 20)	P value IgAN vs. HC	P value DC vs. HC
Oxford classification										
M (M0/M1)	6/12	-			15/35		-	-		
E (E0/E1)	3/15	-			15/35		-	-		
S (S0/S1)	5/13	-			10/40		-	-		
T (T0/T1/T2)	6/6/6	-			28/13/9		33/5/4	-		
C (C0/C1/C2)	2/14/2	-			8/40/2		-	-		
Female (%)	9 (50%)	3 (50%)	> 0.999		23(46%)		22 (52%)	10 (50%)	0.827	0.861
Age ^a^ (years)	34 (29,40)	24 (21,29)	0.172		34(29, 42)		40 (31, 49)	35 (28, 49)	0.131	0.195
Scr ^a^ (µmol/L)	179 (78, 261)	78 (71, 83)	0.119		113 (86, 196)		87 (65, 142)	74(68, 80)	0.034	0.072
eGFR ^a^ (mL/min/1.73m^2^)	37.14 (25.35, 92.32)	109.7 (102.6, 114.0)	0.007		56.9 (30.6, 93.3)		85.6 (33.6, 106.1)	114.3 (106.8, 123.5)	< 0.001	< 0.001
Hemoglobin ^a^ (g/L)	123 (114, 124)	128 (122, 132)	0.172		124 (115, 140)		133 (107, 146)	131 (129, 155)	0.034	0.084
UTP (g/day) ^a^	2.49 (1.12, 3.47)	NA	< 0.001		1.83 (0.80, 2.94)		3.70 (2.36, 6.96)	NA	< 0.001	< 0.001
TG (mmol/L) ^a^	1.76 (1.36, 2.21)	2.11 (1.84, 2.00)	0.424		1.71 (1.07, 2.76)		2.11 (1.48, 3.10)	1.36 (0.97, 2.31)	0.080	0.028
TCHO (mmol/L) ^a^	4.93 (4.01, 5.94)	4.15 (3.93, 4.00)	0.093		4.88 (3.75, 6.02)		5.73 (4.60, 7.62)	4.49 (4.10, 5.25)	0.009	0.009

^a^ median (interquartile range); p values represent the statistical significance between HC and different groups; Scr, Serum Creatinine; eGFR, estimated glomerular filtration rate; UTP, 24-hour urine total protein; TG, triglyceride; TCHO, total cholesterol; NA, not available.

### Proteomics results

**Fig. 1:**
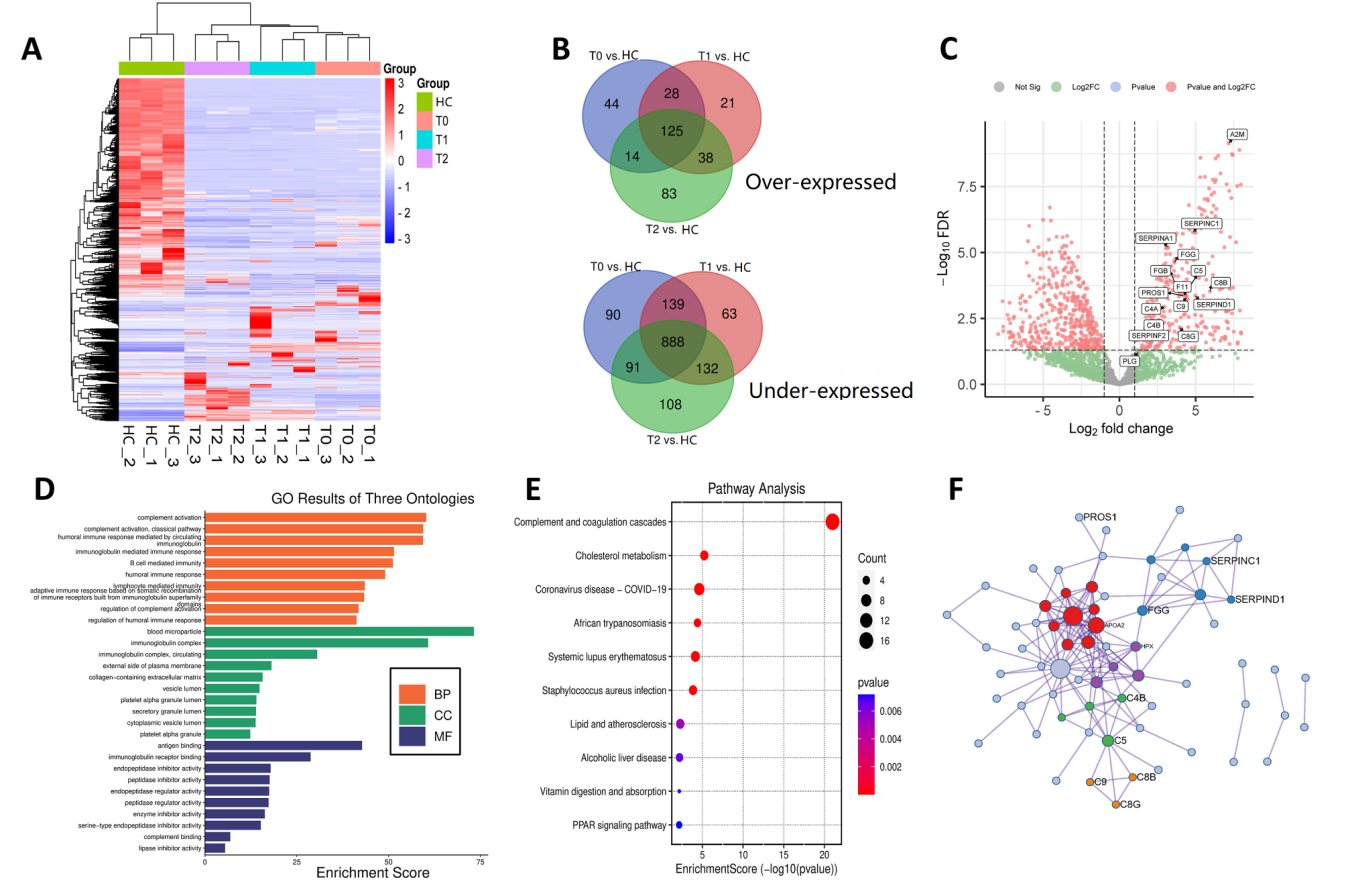
Analysis of differentially expressed proteins. (A) Hierarchical clustering of DEPs in different groups. (B) Venn diagram illustrated the numbers of common DEPs identified by DIA-MS between IgAN and healthy controls. (C) Volcano plots analysis of all DEPs compared to healthy controls. (D) Gene Ontology (GO) enrichment analysis of over-expressed DEPs: Biological Process (BP), Cellular Component (CC) and Molecular Function (MF). (E) Kyoto Encyclopedia of Genes and Genomes (KEGG) pathway analysis of over-expressed DEPs. (F) Protein interaction networks of 125 over-expressed proteins. Proteins related to the extent of renal fibrosis was labeled

Hierarchical clustering shows the expression differences of urinary proteins among the four groups in the discovery cohort (Fig. 1A). Compared to healthy controls, a total of 125 over-expressed and 888 under-expressed urinary proteins were identified in IgAN patients (Fig. 1B-C).

### GO and KEGG pathway annotation and protein interaction network analysis

**Fig. 2:**
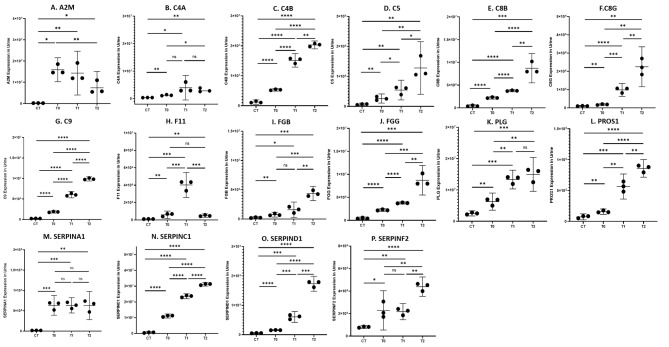
Expressions of 16 DEPs in complement and coagulation cascades signaling pathway (mean with 95% CI). (A-P) Expressions of A2M, C4A, C4B, C5, C8B, C8G, C9, F11, FGB, FGG, HPX, PLG, PROS1, SERPINA1, SERPINC1, SERPIND1 and SERPINF2 in CT, T0, T1 and T2 groups, respectively

We further performed GO analysis and KEGG analysis with the 125 over-expressed proteins (Fig. 1D-E), which were significantly enriched in complement activation process and immunity-related GO terms. It indicated that complement and coagulation cascades pathway (n = 16, *p* < 0.001) was the main enriched KEGG pathway (Table 2). Among the 16 related over-expressed urine proteins (Fig. 2), C4B, C5, C8B, C8G, C9, FGG, PROS1, SERPINC1 and SERPIND1 increased in IgAN patients with the extent of renal fibrosis. However, DIA data of C4B, C5, C8B, PROS1 and SERPIND1 showed no statistical difference between IgAN patients with minimal fibrosis and healthy controls in discovery cohort and C8G showed under-expressed in IgAN patients with minimal fibrosis compared to healthy controls. In the PPI analysis (Fig. 1F), FGG was then considered as a hub protein as it was involved in more pathways (Additional file 3), such as plasma lipoprotein particle clearance (GO:0034381), regulation of blood coagulation (GO:0030193) and scavenging of heme from plasma (R-HSA-2168880). Meanwhile, DIA data showed that C9 was significantly higher in IgAN with 6–25% interstitial fibrosis than those with ≤ 5%. Therefore, FGG and C9 were selected as the potential urinary markers for further validation.

**Table 2:** Differentially expressed proteins in complement and coagulation cascades signaling pathway

No.	Protein ID	Gene Name	Protein Name	Mean Fold Change
1	P01023	A2M	Alpha-2-macroglobulin	70.768
2	P01009	SERPINA1	Alpha-1-antitrypsin	35.883
3	P01008	SERPINC1	Antithrombin-III	35.433
4	P07358	C8B	Complement component C8 beta chain	30.356
5	P02748	C9	Complement component C9	24.819
6	P05546	SERPIND1	Heparin cofactor 2	15.114
7	P03951	F11	Coagulation factor XI	14.752
8	P02679	FGG	Fibrinogen gamma chain	10.972
9	P0C0L5	C4B	Complement C4-B	11.228
10	P07360	C8G	Complement component C8 gamma chain	10.818
11	P02675	FGB	Fibrinogen beta chain	9.722
12	P01031	C5	Complement C5	9.891
13	P0C0L4	C4A	Complement C4-A	7.346
14	P07225	PROS1	Vitamin K-dependent protein S (Fragment)	7.109
15	P00747	PLG	Plasminogen	4.613
16	P08697	SERPINF2	Alpha-2-antiplasmin	3.662

### Increased uFGG with renal fibrosis revealed by ELISA in validation cohort

**Fig. 3:**
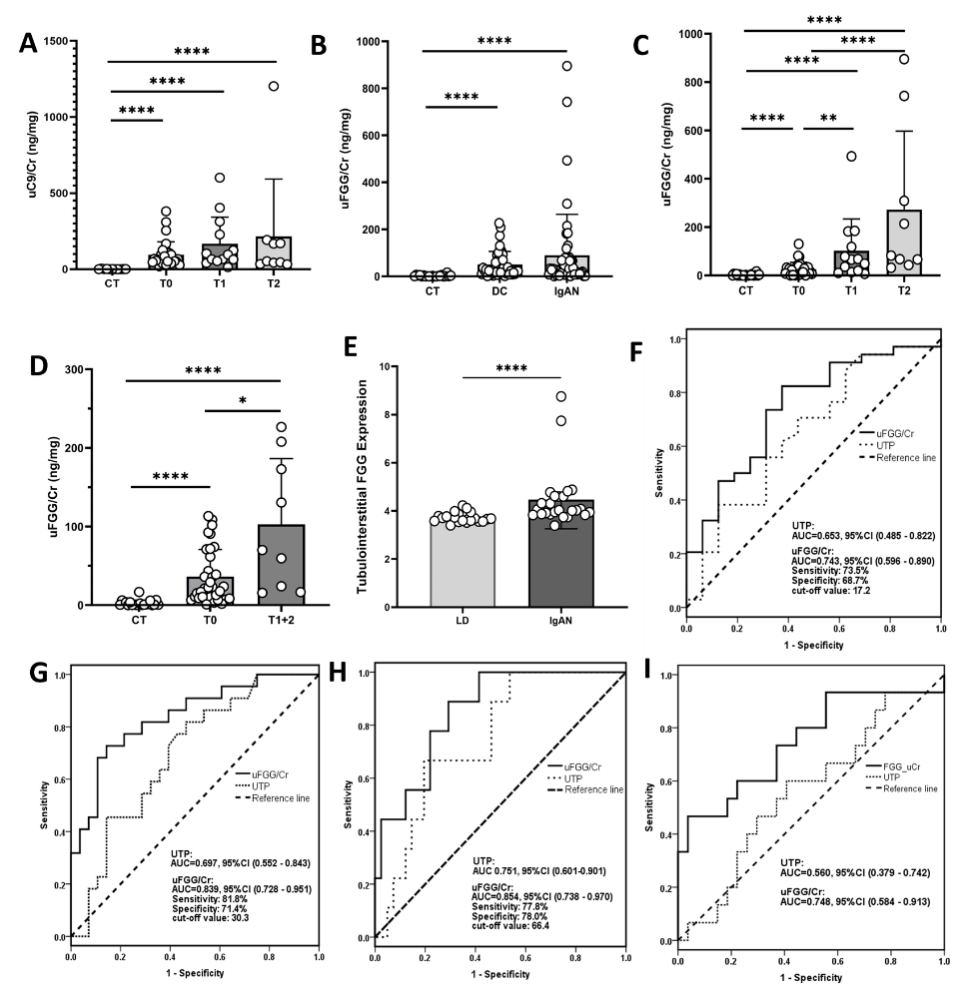
Urinary C9/Cr and FGG/Cr levels in different groups (mean with 95% CI). (A) uC9/Cr levels according to T-scores. (B) uFGG/Cr levels according to T-scores in different groups. (C) uFGG/Cr levels according to T-scores in IgAN patients and healthy controls. (D) uFGG/Cr levels according to T-scores in disease controls and healthy controls. (E) FGG mRNA expression in tubulointerstitium from kidney biopsy of IgAN patients (GSE104954). ROC curves of uFGG/Cr for the discrimination of different groups in IgAN patients between: (F) fibrosis ≤ 5% vs. fibrosis > 5%, (G) T0 vs. T1 + 2, (H) T0 + 1 vs. T2. (I) ROC curves of uFGG/Cr for the discrimination of fibrosis ≤ 5% vs. fibrosis > 5% in disease control patients

We performed ELISA assays for both uFGG and uC9 in an independent validation cohort including 50 biopsy-proven IgAN patients and 20 healthy controls and tested uFGG in 42 disease controls (Additional file 4). Compared to healthy controls, uC9/Cr levels were significantly higher in IgAN patients, but uC9/Cr was not increased with the extent of renal fibrosis (Fig. 3A). uFGG/Cr levels were higher in both disease control group and IgAN group than in healthy controls (Fig. 3B), but were not significantly different between IgAN and disease groups. In IgAN group, uFGG/Cr increased with the extent of renal fibrosis, though there was no statistical difference between T1 and T2 (Fig. 3C). In disease control group, uFGG/Cr was higher in patients with T1 + 2 compared to those with T0 (Fig. 3D). There was no significant correlation between uC9/Cr and T score in IgAN patients, but uFGG/Cr presented moderate correlations with T score (r = 0.614, p < 0.01) and eGFR (r = -0.682, p < 0.01), and a mild correlation with UTP (r = 0.497, p < 0.01) (Table 3).

**Table 3:** Correlations between fibrosis levels and different biomarkers in IgAN group

	T-score	eGFR	UTP	Hgb	uFGG/Cr	uC9/Cr
T-score	1.00	-0.771**	0.376*	-0.367**	0.614**	0.115
eGFR	-	1.00	-0.361**	0.509**	-0.682**	-0.216
UTP	-	-	1.00	-0.375*	0.497**	0.015
Hgb	-	-	-	1.00	-0.480**	-0.162
uFGG/uCr	-	-	-	-	1.00	0.637**
uC9/Cr	-	-	-	-	-	1.00

*: p < 0.05; **: p < 0.01.

### Transcriptomics data analysis

FGG mRNA expression was further analyzed in GSE104954 dataset for transcriptional validation (Additional file 5). Compared to living kidney donors, FGG mRNA was upregulated in tubulointerstitium from kidney biopsy of IgAN patients with a 1.2-fold change (p = 0.009) (Fig. 3E).

### Receiver operator characteristic analysis for advanced fibrosis

ROC curve analysis was performed to discriminate different fibrosis stages with uFGG/Cr. The AUC was 0.743 when < 5% fibrosis was compared to > 5% fibrosis (95% CI: 0.596–0.890) (Fig. 3F). As to T0 vs. T1 + 2 and T0 + 1 vs. T2, uFGG/Cr exhibited good performance in differentiating the earlier fibrosis stages from the advanced (AUC 0.839 and 0.854, respectively) (Fig. 3G-H). uFGG/Cr showed higher AUCs than UTP in IgAN group (Fig. 3F-H), but there was no statistical difference according to DeLong’s test with p value 0.343, 0.061 and 0.116, respectively. In disease control group, uFGG/Cr showed better performance than UTP between minimal and mild fibrosis (p = 0.038 for Delong’s test) (Fig. 3I).

## Discussion

IgAN is a primary type of chronic kidney disease. Studies have shown that fibrosis seen on the renal biopsy of IgAN independently predicted clinical outcome and provided prognostic information [23]. A higher degree of fibrosis means a shorter time for patients to develop ERSD. Renal biopsy is an invasive technique for diagnostic purpose. It is reported that protocol renal biopsies are associated with improved graft survival, but due to its highly invasive nature and the risk of potential complication, they are not routinely performed. [24]. Therefore, there is a need to evaluate renal fibrosis with noninvasive markers. In this study, DIA-MS, a next generation of proteomics technology was used for the first time to reveal that urinary FGG was correlated with the degree of interstitial fibrosis in IgAN.

Over-expressed proteins in urine samples of IgAN patients were found to be primarily enriched in complement and coagulation cascades pathway. It is well established that complement activation contributes to the pathogenesis of IgAN [25]. Virous urinary proteomics studies have indicated this pathway was significantly enriched in IgAN patients [26–29]. Urinary complement proteins were reported to be related with the prognosis or histological findings of IgAN in many studies. Combined urinary biomarkers including fibrinogen alpha and beta chains, which were identified in our study, were shown to present a significant value for the prediction of IgAN progression [29]. Urinary Complement factor H (CFH) was shown to be related with tubular dysfunction and progression of IgAN and urinary C4d correlated with the proportion of crescents in IgAN [30, 31].

Transcriptomic results of a rodent model of nephrotic syndrome disease showed the expressions of hub genes were also enriched in complement and coagulation cascades pathway, suggesting that this pathway is closely related to kidney injury [32]. In fact, the relationship between complement and coagulation cascades pathway and renal fibrosis could be explained. Endothelial cells, the most abundant cells in the capillaries, are the first cells to be damaged by exposure to hemodynamic, immunologic or metabolic injury [33]. The release of tissue factors by damaged endothelial cells triggers the complement and coagulation pathways in innate defense. The related molecules involved in this pathway could deposit in the mesangium, participate in the pathogenesis of renal fibrosis and then present in urine. In this study, 16 over-expressed urinary proteins of complement and coagulation cascades pathway were involved. In the validation group, results demonstrated that uFGG/Cr increased with the degree of fibrosis and correlated moderately with eGFR and Hgb, suggesting its correlation with chronic progression of IgAN. Thus, FGG was suggested as a potential non-invasive urinary biomarker to evaluate interstitial fibrosis in IgAN.

FGG is a primary component of fibrinogen, which is consisted of two α-chains (FGA), two β-chains (FGB), and two γ-chains (FGG) interconnected with disulfide bonds. Fibrinogen is not only synthesized in liver cells, but also in other cells such as epithelial cells or tumor cells [34]. Fibrinogen plays an important role in blood clotting, fibrinolysis, cellular and matrix interactions, inflammatory response and wound healing [35]. In the process of renal fibrosis, fibrinogen can directly stimulate renal fibroblast proliferation in a dose-dependent manner and cooperate with TGF-β1 to induce fibroblast proliferation and activate TGF-β1/PSMAD2 signaling pathway [36, 37]. Roland, et al. [37] utilized a model of unilateral ureteral obstruction in fibrinogen knockout mice and found that compared to fibrinogen deficient homozygous mice (Fib-/-), heterozygous mice (Fib+/-) showed more severe interstitial fibrosis damage, with massive fibrinogen deposition in the renal interstitium. It is worth noting that Wang et al. [38] reported that urinary fibrinogen is an independent risk factor for progression of CKD to ESRD in a study with different types of CKD patients including 152 IgAN patients.

Proteinuria is mainly caused by the damage of glomerular podocytes [39] and it has been shown to be related with progression of IgAN, even at lower levels of UTP (< 1g/d) [40]. It is interesting that uFGG/Cr had a stronger correlation with T score than UTP and AUCs of uFGG/Cr were higher than UTP in IgAN patients, though there was no statistical difference. It also presented higher AUC than UTP between minimal and mild fibrosis in disease control group. These results suggested the superiority of uFGG in renal fibrosis evaluation compared to proteinuria and the possible utility of uFGG for the early evaluation of renal fibrosis. What’s more, uFGG/Cr was higher in patients with T1 + 2 compared to those with T0 in disease control group, suggesting uFGG as a potential universal biomarker of interstitial fibrosis in CKD, but this needs to be confirmed in a large cohort study.

In this study FGG was confirmed to be increased in urine, not necessarily due to a local renal expression. However, transcriptional level of FGG was shown to be increased in the tubulointerstitium from kidney biopsy of IgAN patients in a GEO dataset. Although the transcription level was not always parallel to the protein content, the consistency of FGG mRNA expression in tubulointerstitium and urinary FGG level suggested that FGG might be associated with the process of tubular interstitial fibrosis. As Epithelial-mesenchymal transition (EMT) is considered as an important step of renal fibrosis [41], we propose that, as a primary component of fibrinogen, FGG may also participate in the transition of renal tubular epithelial cells through EMT and promote renal interstitial fibrosis.

There are several limitations in this study. The cohort size is limited, especially for the healthy control population. Further larger sample size studies are needed to reduce the variability of our analyses. We followed up 39 IgAN patients for 12–18 months, but none of them reached an end-point of eGFR decline > 50% or end-stage of kidney disease due to the limited follow-up periods. So, this is a cross-sectional study and further longitudinal studies are needed to assess the prognostic value of uFGG in IgAN and other CKD patients. What’s more, FGG staining was not performed in this study to identify the expression of FGG protein in both renal tubules and interstitium and further studies are needed to confirm the finding.

## Conclusion

In summary, urinary FGG was identified by DIA-MS to be associated with renal interstitial fibrosis in IgAN and was confirmed in a validation cohort in this study. It was concluded that urinary FGG could be used as a non-invasive urinary biomarker for mild renal fibrosis in IgAN. However, large cohort studies and mechanistic investigations are required for further validation.

## Electronic supplementary material

Below is the link to the electronic supplementary material.

Additional file 1

Additional file 2

Additional file 3

Additional file 4

Additional file 5

## Data Availability

Publicly available dataset was analyzed in this study and these data can be found here: https://www.ncbi.nlm.nih.gov/geo/, GSE104954. The experimental data used and analyzed in the current study were available from the supplementary material.

## List of abbreviations

IgAN: IgA nephropathy
CKD: chronic kidney disease
ESRD: end-stage renal disease
ECM: extracellular matrix
EGF: epidermal growth factor
LC-MS/MS: liquid chromatography with tandem mass spectrometry
CE-MS: capillary electrophoresis coupled to mass spectrometry
DIA-MS: data independent acquisition-based liquid chromatography-mass spectrometry
HC: healthy control
eGFR: estimated glomerular filtration rate
Hgb: hemoglobin
ELISA: enzyme-linked immunosorbent assay
ESI: electrospray ionization
FA: formic acid
AGC: autogain control
GEO: Gene Expression Omnibus
FDR: false discovery rate
FC: fold change
DEP: differentially expressed protein
GO: Gene Ontology
KEGG: Kyoto Encyclopedia of Genes and Genomes
PPI: protein‑protein interaction
IQR: interquartile range
ROC: receiver operating characteristics curves
AUC: area under the curve
MCD: minimal change disease
DC: disease control
DN: diabetic nephropathy
MN: membranous nephropathy
BP: biological process
CC: cellular component
MF: molecular function
FGG: fibrinogen γ-Chain
FAG: fibrinogen α-chains
FBG: fibrinogen β-chains
C9: complement C9
Cr: creatinine
CFH: complement factor H
EMT: epithelial-mesenchymal transition

## References

[1] Li L-S, Liu Z-H (2004). Epidemiologic data of renal diseases from a single unit in China: analysis based on 13,519 renal biopsies. Kidney Int.

[2] Pattrapornpisut P, Avila-Casado C, Reich HN (2021). IgA Nephropathy: Core Curriculum 2021. Am J Kidney Dis.

[3] Yang M, Liu JW, Zhang YT, Wu G (2021). The role of renal macrophage, AIM, and TGF-beta1 expression in Renal Fibrosis Progression in IgAN Patients. Front Immunol.

[4] Zhang H, Barratt J (2021). Is IgA nephropathy the same disease in different parts of the world?. Semin Immunopathol.

[5] Coppo R, D’Arrigo G, Tripepi G, Russo ML, Roberts ISD, Bellur S (2020). Is there long-term value of pathology scoring in immunoglobulin A nephropathy? A validation study of the Oxford classification for IgA Nephropathy (VALIGA) update. Nephrol Dial Transplant.

[6] Xu R, Li Z, Cao T, Xu Y, Liao Y, Song H (2021). The Association of the Oxford classification score with longitudinal estimated glomerular filtration rate decline in patients with immunoglobulin A nephropathy: a mixed-method study. Int J Gen Med.

[7] Wu H, Xia Z, Gao C, Zhang P, Yang X, Wang R (2020). The correlation analysis between the Oxford classification of chinese IgA nephropathy children and renal outcome - a retrospective cohort study. BMC Nephrol.

[8] Humphreys BD (2018). Mechanisms of Renal Fibrosis. Annu Rev Physiol.

[9] Mavrogeorgis E, Mischak H, Beige J, Latosinska A, Siwy J (2021). Understanding glomerular diseases through proteomics. Expert Rev Proteomics.

[10] Zhou LT, Lv LL, Liu BC (2019). Urinary biomarkers of Renal Fibrosis. Adv Exp Med Biol.

[11] Chebotareva N, Vinogradov A, McDonnell V, Zakharova NV, Indeykina MI, Moiseev S (2021). Urinary protein and peptide markers in chronic kidney disease. Int J Mol Sci.

[12] Mejia-Vilet JM, Shapiro JP, Zhang XL, Cruz C, Zimmerman G, Mendez-Perez RA (2021). Association between urinary epidermal growth factor and renal prognosis in Lupus Nephritis. Arthritis Rheumatol.

[13] Magalhaes P, Pejchinovski M, Markoska K, Banasik M, Klinger M, Svec-Billa D (2017). Association of kidney fibrosis with urinary peptides: a path towards non-invasive liquid biopsies?. Sci Rep.

[14] Catanese L, Siwy J, Mavrogeorgis E, Amann K, Mischak H, Beige J (2021). A novel urinary proteomics classifier for non-invasive evaluation of interstitial fibrosis and tubular atrophy in chronic kidney disease. Proteomes.

[15] Tailliar M, Schanstra J, Dierckx T, Breuil B, Hanouna G, Charles N (2021). Urinary peptides as potential non-invasive biomarkers for Lupus Nephritis: results of the Peptidu-LUP Study. J Clin Med.

[16] Argiles A, Siwy J, Duranton F, Gayrard N, Dakna M, Lundin U (2013). CKD273, a new proteomics classifier assessing CKD and its prognosis. PLoS ONE.

[17] Schanstra JP, Zurbig P, Alkhalaf A, Argiles A, Bakker SJ, Beige J (2015). Diagnosis and prediction of CKD progression by Assessment of urinary peptides. J Am Soc Nephrol.

[18] Levey AS, Stevens LA, Schmid CH, Zhang YL, Castro AF 3rd, Feldman HI, et al. A new equation to estimate glomerular filtration rate. Ann Intern Med. 2009;150(9):604–12. 10.7326/0003-4819-150-9-200905050-00006 PMC2763564 19414839

[19] Sparding N, Genovese F, Rasmussen DGK, Karsdal MA, Neprasova M, Maixnerova D (2022). Endotrophin, a collagen type VI-derived matrikine, reflects the degree of renal fibrosis in patients with IgA nephropathy and in patients with ANCA-associated vasculitis. Nephrol Dial Transplant.

[20] Metsalu T, Vilo J (2015). ClustVis: a web tool for visualizing clustering of multivariate data using principal component analysis and heatmap. Nucleic Acids Res.

[21] Wu T, Hu E, Xu S, Chen M, Guo P, Dai Z (2021). clusterProfiler 4.0: a universal enrichment tool for interpreting omics data. Innov (Camb).

[22] Kanehisa M, Furumichi M, Sato Y, Kawashima M, Ishiguro-Watanabe M (2023). KEGG for taxonomy-based analysis of pathways and genomes. Nucleic Acids Res.

[23] Trimarchi H, Barratt J, Cattran DC, Cook HT, Coppo R, Haas M (2017). Oxford classification of IgA nephropathy 2016: an update from the IgA nephropathy classification Working Group. Kidney Int.

[24] Terrec F, Noble J, Naciri-Bennani H, Malvezzi P, Janbon B, Emprou C (2021). Protocol biopsies on de novo renal-transplants at 3 months after surgery: impact on 5-Year transplant survival. J Clin Med.

[25] Medjeral-Thomas NR, Cook HT, Pickering MC (2021). Complement activation in IgA nephropathy. Semin Immunopathol.

[26] Fang X, Lu M, Xia Z, Gao C, Cao Y, Wang R (2021). Use of liquid chromatography-tandem mass spectrometry to perform urinary proteomic analysis of children with IgA nephropathy and Henoch-Schonlein purpura nephritis. J Proteom.

[27] Guo Z, Wang Z, Lu C, Yang S, Sun H, Reziw (2018). Analysis of the differential urinary protein profile in IgA nephropathy patients of Uygur ethnicity. BMC Nephrol.

[28] Samavat S, Kalantari S, Nafar M, Rutishauser D, Rezaei-Tavirani M, Parvin M (2015). Diagnostic urinary proteome profile for immunoglobulin a nephropathy. Iran J Kidney Dis.

[29] Rudnicki M, Siwy J, Wendt R, Lipphardt M, Koziolek MJ, Maixnerova D (2021). Urine proteomics for prediction of disease progression in patients with IgA nephropathy. Nephrol Dial Transplant.

[30] Wen L, Zhao Z, Wang Z, Xiao J, Birn H, Gregersen JW (2019). High levels of urinary complement proteins are associated with chronic renal damage and proximal tubule dysfunction in immunoglobulin A nephropathy. Nephrol (Carlton).

[31] Liu M, Chen Y, Zhou J, Liu Y, Wang F, Shi S (2015). Implication of urinary complement factor H in the progression of immunoglobulin A nephropathy. PLoS ONE.

[32] He S, Li A, Zhang W, Zhang L, Liu Y, Li K (2020). An integrated transcriptomics and network pharmacology approach to exploring the mechanism of adriamycin-induced kidney injury. Chem Biol Interact.

[33] Trimarchi H, Coppo R (2021). Glomerular endothelial activation, C4d deposits and microangiopathy in immunoglobulin A nephropathy. Nephrol Dial Transplant.

[34] Peng HH, Wang JN, Xiao LF, Yan M, Chen SP, Wang L (2021). Elevated serum FGG levels Prognosticate and promote the Disease progression in prostate Cancer. Front Genet.

[35] Mosesson MW (2005). Fibrinogen and fibrin structure and functions. J Thromb Haemost.

[36] Craciun FL, Ajay AK, Hoffmann D, Saikumar J, Fabian SL, Bijol V (2014). Pharmacological and genetic depletion of fibrinogen protects from kidney fibrosis. Am J Physiol Renal Physiol.

[37] Sorensen I, Susnik N, Inhester T, Degen JL, Melk A, Haller H (2011). Fibrinogen, acting as a mitogen for tubulointerstitial fibroblasts, promotes renal fibrosis. Kidney Int.

[38] Wang H, Zheng C, Lu Y, Jiang Q, Yin R, Zhu P (2017). Urinary fibrinogen as a predictor of progression of CKD. Clin J Am Soc Nephrol.

[39] Torban E, Braun F, Wanner N, Takano T, Goodyer PR, Lennon R (2019). From podocyte biology to novel cures for glomerular disease. Kidney Int.

[40] Habas E, Ali E, Farfar K, Errayes M, Alfitori J, Habas E (2022). IgA nephropathy pathogenesis and therapy: Review & updates. Med (Baltim).

[41] Sun YB, Qu X, Caruana G, Li J (2016). The origin of renal fibroblasts/myofibroblasts and the signals that trigger fibrosis. Differentiation.

