# Supplementary figures and images for: Discovery of Fibrinogen γ-chain as a potential urinary biomarker for renal interstitial fibrosis in IgA nephropathy
Source: BMC Nephrol. 2023 Mar 20;24:60. doi: 10.1186/s12882-023-03103-7 (PMC10029243; doi:10.1186/s12882-023-03103-7)

Supplementary figures legend


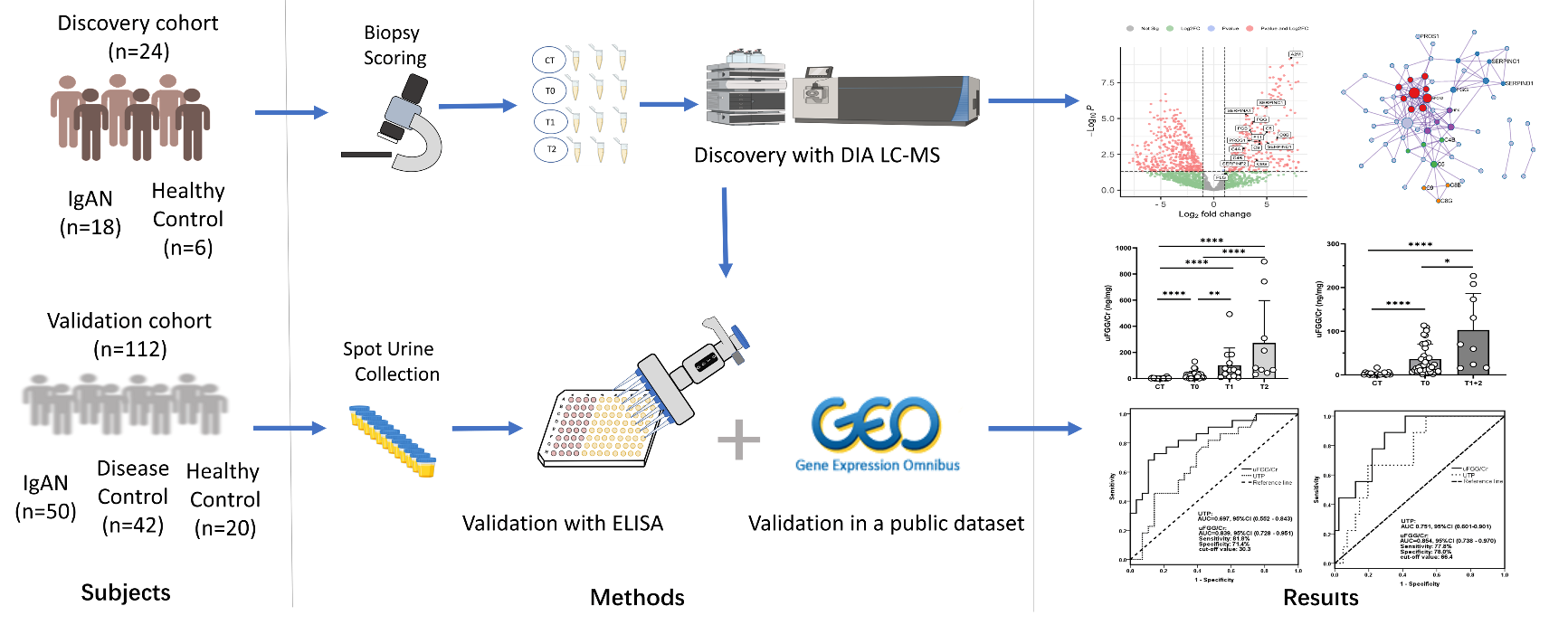
Additional file 1. Graphical abstract.

Supplement: Supplementary file 1 — Additional file 1 [file 12882_2023_3103_MOESM1_ESM.docx]
