# Supplementary material for: Discovery of Fibrinogen γ-chain as a potential urinary biomarker for renal interstitial fibrosis in IgA nephropathy
Source: BMC Nephrol. 2023 Mar 20;24:60. doi: 10.1186/s12882-023-03103-7 (PMC10029243; doi:10.1186/s12882-023-03103-7)
Supplement: Supplementary file 2 — Additional file 2 [file 12882_2023_3103_MOESM2_ESM.docx]

Supplementary file legend

Additional 2. Raw data of clinical characteristics and baseline biopsy evaluation in discovery and validation set.

| sID | gender | age | | M | | E | | S | | T | | C | | CKD | | Hbg | | Scr | | e-GFR | | UTP | | TG | | TCHO | |  |
| --- | --- | --- | --- | --- | --- | --- | --- | --- | --- | --- | --- | --- | --- | --- | --- | --- | --- | --- | --- | --- | --- | --- | --- | --- | --- | --- | --- | --- |
| 20072008 | 1 | 21 | | 1 | | 1 | | 1 | | 0 | | 2 | | 1 | | 150.00 | | 81.1 | | 118.874 | | 1.05 | | 0.87 | | 3.79 | |  |
| 20071601 | 2 | 27 | | 0 | | 0 | | 0 | | 0 | | 1 | | 1 | | 117.00 | | 76.4 | | 92.324 | | 0.12 | | 0.49 | | 4.57 | |  |
| 20073001 | 1 | 29 | | 1 | | 1 | | 1 | | 0 | | 1 | | 1 | | 111.00 | | 72.3 | | 97.314 | | 2.82 | | 1.6 | | 6.53 | |  |
| 20060813 | 2 | 32 | | 1 | | 1 | | 1 | | 0 | | 1 | | 1 | | 124.00 | | 60.6 | | 115.804 | | 4.67 | | 1.35 | | 4.99 | |  |
| 20060104 | 1 | 34 | | 1 | | 1 | | 1 | | 0 | | 1 | | 2 | | 155.00 | | 125.47 | | 64.014 | | 0.64 | | 1.38 | | 5.08 | |  |
| 20071606 | 2 | 41 | | 0 | | 1 | | 0 | | 0 | | 1 | | 1 | | 116.00 | | 63.74 | | 104.167 | | 2.15 | | 2.21 | | 6.58 | |  |
| 20060405 | 2 | 28 | | 1 | | 1 | | 1 | | 1 | | 1 | | 3 | | 75.00 | | 174.2 | | 33.845 | | 2.94 | | 2.58 | | 6.76 | |  |
| 20042301 | 2 | 29 | | 0 | | 0 | | 0 | | 1 | | 1 | | 1 | | 122.00 | | 68.5 | | 103.878 | | 0.30 | | 0.87 | | 4.5 | |  |
| 20060801 | 1 | 34 | | 1 | | 1 | | 1 | | 1 | | 1 | | 3 | | 130.00 | | 183.5 | | 40.429 | | 3.4 | | 1.91 | | 5.94 | |  |
| 20041303 | 1 | 38 | | 1 | | 1 | | 1 | | 1 | | 0 | | 3 | | 115.00 | | 144.05 | | 52.673 | | 0.78 | | 2.01 | | 3.22 | |  |
| 20050702 | 2 | 44 | | 1 | | 1 | | 0 | | 1 | | 2 | | 4 | | 124.00 | | 202.63 | | 25.195 | | 6.87 | | 4.21 | | 4.79 | |  |
| 20033001 | 1 | 65 | | 0 | | 0 | | 1 | | 1 | | 1 | | 3 | | 123.0 | | 194.41 | | 30.325 | | 3.22 | | 3.29 | | 4.87 | |  |
| 20060808 | 1 | 30 | | 0 | | 1 | | 1 | | 2 | | 1 | | 4 | | 123.00 | | 322.6 | | 21.021 | | 1.44 | | 3.92 | | 5.75 | |  |
| 20072001 | 2 | 31 | | 1 | | 1 | | 1 | | 2 | | 1 | | 5 | | 113.00 | | 809.8 | | 5.171 | | 3.47 | | 1.61 | | 3.85 | |  |
| 20072005 | 1 | 37 | | 0 | | 1 | | 1 | | 2 | | 1 | | 4 | | 139.00 | | 261.4 | | 25.808 | | 1.34 | | 1.95 | | 5.22 | |  |
| 20041304 | 2 | 38 | | 1 | | 1 | | 1 | | 2 | | 1 | | 5 | | 96.00 | | 643.7 | | 6.497 | | 5.04 | | 1.49 | | 6.46 | |  |
| 20081302 | 1 | 42 | | 1 | | 1 | | 0 | | 2 | | 1 | | 3 | | 124.00 | | 215.86 | | 31.406 | | 1.74 | | 0.94 | | 2.18 | |  |
| 20050706 | 2 | 46 | | 1 | | 1 | | 1 | | 2 | | 0 | | 5 | | 44.00 | | 434.69 | | 9.873 | | 4.13 | | 2.11 | | 2.67 | |  |
| UCTF2004 | 2 | 19 | | * | | * | | * | | * | | * | | * | | * | | * | | * | | * | | * | | * | |  |
| UCTM2002 | 1 | 21 | | * | | * | | * | | * | | * | | * | | * | | * | | * | | * | | * | | * | |  |
| UCTM2003 | 1 | 21 | | * | | * | | * | | * | | * | | * | | * | | * | | * | | * | | * | | * | |  |
| UCTF2006 | 2 | 27 | | * | | * | | * | | * | | * | | * | | * | | * | | * | | * | | * | | * | |  |
| UCTF2005 | 2 | 29 | | * | | * | | * | | * | | * | | * | | * | | * | | * | | * | | * | | * | |  |
| UCTM2001 | 1 | 32 | | * | | * | | * | | * | | * | | * | | * | | * | | * | | * | | * | | * | |  |
|  |  |  | |  | |  | |  | |  | |  | |  | |  | |  | |  | |  | |  | |  | |  |
|  |  |  | |  | |  | |  | |  | |  | |  | |  | |  | |  | |  | |  | |  | |  |
| IgAN_median | | | 34 | |  | |  | |  | |  | |  | |  | | 122.50 | | 178.85 | | 37.14 | | 2.49 | | 1.76 | | 4.93 | |
| IQR1 |  | 29 | |  | |  | |  | |  | |  | |  | | 113.50 | | 77.58 | | 25.35 | | 1.12 | | 1.36 | | 4.01 | |  |
| IQR3 |  | 40 | |  | |  | |  | |  | |  | |  | | 124.00 | | 261.40 | | 92.32 | | 3.47 | | 2.21 | | 5.94 | |  |
|  |  |  | |  | |  | |  | |  | |  | |  | |  | |  | |  | |  | |  | |  | |  |
| HC_median | | | 24 | |  | |  | |  | |  | |  | |  | |  | |  | |  | |  | |  | |  | |
| IQR1 |  | 21 | |  | |  | |  | |  | |  | |  | |  | |  | |  | |  | |  | |  | |  |
| IQR3 |  | 29 | |  | |  | |  | |  | |  | |  | |  | |  | |  | |  | |  | |  | |  |
|  |  |  | |  | |  | |  | |  | |  | |  | |  | |  | |  | |  | |  | |  | |  |
