# Supplementary material for: Discovery of Fibrinogen γ-chain as a potential urinary biomarker for renal interstitial fibrosis in IgA nephropathy
Source: BMC Nephrol. 2023 Mar 20;24:60. doi: 10.1186/s12882-023-03103-7 (PMC10029243; doi:10.1186/s12882-023-03103-7)

Supplementary file legend

Additional file 3. Pathways and process enrichment of over-expressed proteins with the Molecular Complex Detection (MCODE) algorithm.

| MCODE | GO | Description | Log10(P) |
| --- | --- | --- | --- |
| MCODE_1 | GO:0034381 | plasma lipoprotein particle clearance | -17 |
| MCODE_1 | GO:0097006 | regulation of plasma lipoprotein particle levels | -14.6 |
| MCODE_1 | R-HSA-8963898 | Plasma lipoprotein assembly | -14.2 |
| MCODE_2 | hsa04610 | Complement and coagulation cascades | -15.4 |
| MCODE_2 | WP558 | Complement and coagulation cascades | -12.9 |
| MCODE_2 | GO:0030193 | regulation of blood coagulation | -12.5 |
| MCODE_3 | R-HSA-174577 | Activation of C3 and C5 | -10.3 |
| MCODE_3 | WP545 | Complement activation | -8.8 |
| MCODE_3 | GO:0010951 | negative regulation of endopeptidase activity | -8.3 |
| MCODE_4 | R-HSA-2168880 | Scavenging of heme from plasma | -9.6 |
| MCODE_4 | R-HSA-2173782 | Binding and Uptake of Ligands by Scavenger Receptors | -8 |
| MCODE_4 | R-HSA-5653656 | Vesicle-mediated transport | -4.4 |
| MCODE_5 | R-HSA-166665 | Terminal pathway of complement | -10.9 |
| MCODE_5 | GO:0006957 | complement activation, alternative pathway | -10 |
| MCODE_5 | GO:0019835 | cytolysis | -9.5 |


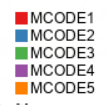

Supplement: Supplementary file 3 — Additional file 3 [file 12882_2023_3103_MOESM3_ESM.docx]
