# Supplementary material for: Discovery of Fibrinogen γ-chain as a potential urinary biomarker for renal interstitial fibrosis in IgA nephropathy
Source: BMC Nephrol. 2023 Mar 20;24:60. doi: 10.1186/s12882-023-03103-7 (PMC10029243; doi:10.1186/s12882-023-03103-7)
Supplement: Supplementary file 4 — Additional file 4 [file 12882_2023_3103_MOESM4_ESM.docx]

Supplementary file legend

Additional file 4. Raw data of clinical parameters, urinary C9 and FGG results in validation cohort.

| NO. | sID | gender | age | Group | M | E | S | T | C | T0 | Hbg | Scr | e-GFR | TG | TCHO | UTP | C9_cr | FGG_cr |
| --- | --- | --- | --- | --- | --- | --- | --- | --- | --- | --- | --- | --- | --- | --- | --- | --- | --- | --- |
| 1 | 20033001 | 1 | 65 | IgAN | 0 | 0 | 1 | 1 | 1 | moderate | 123 | 194.41 | 30.325 | 3.29 | 4.87 | 3.22 | 64.01 | 49.22 |
| 2 | 20040703 | 2 | 30 | IgAN | 1 | 1 | 1 | 2 | 1 | severe | 92 | 419.4 | 11.536 | 1.15 | 6.14 | 7.96 | 43.56 | 309.13 |
| 3 | 20041306 | 1 | 18 | IgAN | 0 | 1 | 1 | 0 | 1 | minimal | 140 | 64.2 | 105.179 | 2.31 | 3.71 | 1.01 | 96.10 | 42.17 |
| 4 | 20041303 | 1 | 38 | IgAN | 1 | 1 | 1 | 1 | 0 | moderate | 115 | 144.05 | 52.673 | 2.01 | 3.22 | 0.78 | 14.00 | 7.20 |
| 5 | 20041304 | 2 | 38 | IgAN | 1 | 1 | 1 | 2 | 1 | severe | 96 | 643.7 | 6.497 | 1.49 | 6.46 | 5.04 | 168.27 | 742.77 |
| 6 | 20042302 | 2 | 37 | IgAN | 1 | 1 | 1 | 0 | 1 | minimal | 135 | 87.8 | 72.742 | 1 | 5.36 | 1.39 | 63.07 | 33.82 |
| 7 | 20042301 | 2 | 29 | IgAN | 0 | 0 | 0 | 0 | 1 | minimal | 122 | 68.5 | 103.878 | 0.87 | 4.5 | 0.3 | 309.87 | 11.74 |
| 8 | 20050702 | 2 | 44 | IgAN | 1 | 1 | 0 | 1 | 2 | moderate | 124 | 202.63 | 25.195 | 4.21 | 4.79 | 6.87 | 96.50 | 493.30 |
| 9 | 20050706 | 2 | 46 | IgAN | 1 | 1 | 1 | 2 | 0 | severe | 44 | 434.69 | 9.873 | 2.11 | 2.67 | 4.13 | 1202.39 | 895.70 |
| 10 | 20052602 | 1 | 25 | IgAN | 1 | 1 | 0 | 2 | 1 | severe | 115 | 369.58 | 18.473 | 3.01 | 4.58 | 2.92 | 45.88 | 66.58 |
| 11 | 20060103 | 2 | 32 | IgAN | 0 | 1 | 1 | 0 | 1 | minimal | 132 | 81.68 | 82.22 | 2.24 | 3.54 | 0.64 | 30.00 | 0.87 |
| 12 | 20060104 | 1 | 34 | IgAN | 1 | 1 | 1 | 1 | 1 | moderate | 155 | 125.47 | 64.014 | 1.38 | 5.08 | 0.64 | 103.42 | 13.71 |
| 13 | 20060405 | 2 | 28 | IgAN | 1 | 1 | 1 | 2 | 1 | severe | 75 | 174.2 | 33.845 | 2.58 | 6.76 | 2.94 | 53.19 | 42.89 |
| 14 | 20060803 | 2 | 33 | IgAN | 0 | 0 | 0 | 0 | 1 | minimal | 128 | 60.1 | 115.308 | 0.88 | 5.73 | 1.07 | 27.92 | 1.37 |
| 15 | 20060813 | 2 | 32 | IgAN | 1 | 1 | 1 | 0 | 1 | mild | 124 | 60.6 | 115.804 | 1.35 | 4.99 | 4.67 | 37.70 | 21.78 |
| 16 | 20060801 | 1 | 34 | IgAN | 1 | 1 | 1 | 1 | 1 | moderate | 130 | 183.5 | 40.429 | 1.91 | 5.94 | 3.4 | 53.29 | 19.71 |
| 17 | 20060808 | 1 | 30 | IgAN | 0 | 1 | 1 | 2 | 1 | severe | 123 | 322.6 | 21.021 | 3.92 | 5.75 | 1.44 | 192.54 | 82.07 |
| 18 | 20071601 | 2 | 27 | IgAN | 0 | 0 | 0 | 0 | 1 | mild | 117 | 76.4 | 92.324 | 0.49 | 4.57 | 0.12 | 380.53 | 13.50 |
| 19 | 20071606 | 2 | 41 | IgAN | 0 | 1 | 0 | 0 | 1 | mild | 116 | 63.74 | 104.167 | 2.21 | 6.58 | 2.15 | 53.04 | 10.56 |
| 20 | 20072008 | 1 | 21 | IgAN | 1 | 1 | 1 | 0 | 2 | mild | 150 | 81.1 | 118.874 | 0.87 | 3.79 | 1.05 | 52.77 | 28.32 |
| 21 | 20072001 | 2 | 31 | IgAN | 1 | 1 | 1 | 2 | 1 | severe | 113 | 809.8 | 5.171 | 1.61 | 3.85 | 3.47 | 170.15 | 214.35 |
| 22 | 20072005 | 1 | 37 | IgAN | 0 | 1 | 1 | 2 | 1 | severe | 139 | 261.4 | 25.808 | 1.95 | 5.22 | 1.34 | 32.56 | 32.26 |
| 23 | 20073001 | 1 | 29 | IgAN | 1 | 1 | 1 | 0 | 1 | mild | 111 | 72.3 | 97.314 | 1.6 | 6.53 | 2.82 | 125.49 | 39.04 |
| 24 | 20073002 | 2 | 39 | IgAN | 1 | 0 | 1 | 0 | 0 | mild | 130 | 58.35 | 111.629 | 2.53 | 6.33 | 1.37 | 83.05 | 14.71 |
| 25 | 20073003 | 1 | 56 | IgAN | 0 | 0 | 1 | 0 | 0 | mild | 118 | 111 | 47.941 |  |  | 0.73 | 168.29 | 81.48 |
| 26 | 20080302 | 2 | 34 | IgAN | 1 | 1 | 1 | 0 | 1 | minimal | 113 | 73.4 | 92.256 | 2.09 | 5.45 | 2.33 | 19.66 | 7.14 |
| 27 | 20081002 | 1 | 18 | IgAN | 1 | 1 | 1 | 0 | 1 | minimal | 150 | 74.4 | 83.401 | 0.8 | 2.92 | 0.34 | 55.96 | 8.31 |
| 28 | 20081003 | 1 | 20 | IgAN | 1 | 1 | 1 | 0 | 1 | minimal | 142 | 86.1 | 111.359 | 1.44 | 5.13 | 2.58 | 37.70 | 12.72 |
| 29 | 20081302 | 1 | 42 | IgAN | 1 | 1 | 0 | 2 | 1 | severe | 124 | 215.86 | 31.406 | 0.94 | 2.18 | 1.74 | 34.19 | 67.31 |
| 30 | 20081705 | 1 | 36 | IgAN | 1 | 1 | 1 | 0 | 1 | mild | 162 | 150.16 | 50.802 | 8.46 | 3.57 | 0.7 | 95.79 | 8.82 |
| 31 | 20082408 | 1 | 20 | IgAN | 1 | 1 | 1 | 0 | 1 | minimal | 139 | 85.34 | 112.559 |  |  | 3.52 | 46.32 | 13.31 |
| 32 | 20082701 | 2 | 42 | IgAN | 0 | 1 | 1 | 0 | 1 | minimal | 119 | 127.6 | 44.693 | 1.11 | 4.88 | 1.91 | 66.93 | 47.51 |
| 33 | 20082704 | 2 | 55 | IgAN | 0 | 0 | 1 | 0 | 1 | minimal | 100 | 96.8 | 56.968 | 3.43 | 8.09 | 0.58 | 123.04 | 76.93 |
| 34 | 21081401 | 1 | 22 | IgAN | 1 | 0 | 1 | 0 | 1 | minimal | 151 | 94.7 | 97.867 | 0.98 | 4.76 | 0.44 | 51.88 | 1.95 |
| 35 | 21081407 | 2 | 62 | IgAN | 1 | 1 | 1 | 0 | 1 | mild | 116 | 140.9 | 34.44 | 2.95 | 8.18 | 14.09 | 104.83 | 32.50 |
| 36 | 21082005 | 1 | 42 | IgAN | 1 | 1 | 1 | 0 | 0 | mild | 170 | 87.5 | 93.571 | 1.03 | 3.67 | 0.41 | 85.72 | 0.76 |
| 37 | 21082006 | 2 | 50 | IgAN | 1 | 1 | 0 | 0 | 1 | minimal | 124 | 103.97 | 54.121 | 4.7 | 7.71 | 9.43 | 254.30 | 130.47 |
| 38 | 21082303 | 2 | 33 | IgAN | 1 | 1 | 1 | 1 | 1 | moderate | 155 | 89 | 73.597 | 1.38 | 5.08 | 0.64 | 228.55 | 182.19 |
| 39 | 21082308 | 2 | 53 | IgAN | 1 | 0 | 1 | 1 | 0 | moderate | 77 | 196 | 24.622 |  |  | 1.06 | 65.52 | 36.47 |
| 40 | 21083001 | 1 | 52 | IgAN | 0 | 1 | 1 | 1 | 1 | moderate | 130 | 290.4 | 20.453 | 5.25 | 2.82 | 1.97 | 41.04 | 184.34 |
| 41 | 21083004 | 2 | 37 | IgAN | 0 | 1 | 1 | 1 | 1 | moderate | 80 | 530.7 | 8.263 | 3.19 | 4.38 | 2.31 | 404.08 | 78.32 |
| 42 | 21090207 | 1 | 20 | IgAN | 1 | 1 | 1 | 0 | 1 | minimal | 143 | 107.3 | 85.339 | 0.7 | 4.3 | 2.32 | 47.82 | 8.04 |
| 43 | 21090209 | 1 | 68 | IgAN | 1 | 0 | 0 | 0 | 0 | minimal | 129 | 157.66 | 38.253 | 1.24 | 4.97 | 1.32 | 60.46 | 14.72 |
| 44 | 21090214 | 1 | 36 | IgAN | 1 | 1 | 0 | 0 | 1 | minimal | 123 | 96.7 | 86.487 | 1.71 | 6.17 | 0.24 | 69.00 | 4.92 |
| 45 | 21090215 | 1 | 19 | IgAN | 1 | 0 | 1 | 0 | 1 | mild | 150 | 90.1 | 106.153 | 1.37 | 4.04 | 2.04 | 53.81 | 3.21 |
| 46 | 21090218 | 1 | 31 | IgAN | 1 | 1 | 1 | 0 | 1 | mild | 137 | 141.03 | 56.763 | 0.61 | 1.95 | 0.87 | 45.40 | 27.91 |
| 47 | 21090902 | 2 | 32 | IgAN | 1 | 0 | 1 | 1 | 1 | moderate | 117 | 102.41 | 62.549 | 1.85 | 6.1 | 2.11 | 313.47 | 68.28 |
| 48 | 21090903 | 1 | 64 | IgAN | 0 | 0 | 1 | 1 | 0 | moderate | 148 | 114.93 | 57.656 | 3.53 | 3.64 | 4.9 | 601.84 | 66.12 |
| 49 | 21090909 | 1 | 33 | IgAN | 1 | 0 | 1 | 1 | 1 | moderate | 86 | 986.45 | 5.329 | 0.93 | 3.59 | 2.31 | 65.36 | 118.26 |
| 50 | 21090911 | 1 | 64 | IgAN | 1 | 0 | 1 | 1 | 1 | moderate | 144 | 147.23 | 42.737 | 2.93 | 6.16 | 1.39 | 123.93 | 11.10 |
| 51 | 2449168 | 2 | 49 | DN | - | - | - | 0 | - | - | 81 | 204.79 | 23.514 | 2.44 | 4.58 | 2.21 | NA | 113.29 |
| 52 | 2438575 | 2 | 26 | DN | - | - | - | 0 | - | - | 137 | 60.1 | 121.119 | 0.8 | 3.5 | 1.55 | NA | 10.45 |
| 53 | 2442744 | 1 | 50 | DN | - | - | - | 0 | - | - | 165 | 100.1 | 73.611 | 1.3 | 4.6 | 3.25 | NA | 15.18 |
| 54 | 2442367 | 2 | 58 | DN | - | - | - | 0 | - | - | 88 | 120.2 | 42.038 | 1.46 | 5.4 | 5.95 | NA | 92.75 |
| 55 | 2443868 | 1 | 55 | DN | - | - | - | 0 | - | - | 113 | 498.41 | 10.205 | 0.84 | 5.75 | 2.54 | NA | 22.61 |
| 56 | 2442969 | 2 | 25 | DN | - | - | - | 1 | - | - | 96 | 113.37 | 58.102 | 1.69 | 7.79 | 7.04 | NA | 172.96 |
| 57 | 2444035 | 1 | 40 | DN | - | - | - | 1 | - | - | 109 | 257.4 | 22.059 | 1.23 | 3.93 | 1.92 | NA | 24.07 |
| 58 | 2446942 | 1 | 40 | DN | - | - | - | 1 | - | - | 85 | 432.07 | 13.477 | 1.71 | 3.25 | 3.2 | NA | 59.59 |
| 59 | 2403331 | 1 | 58 | DN | - | - | - | 2 | - | - | 98 | 369.8 | 14.335 | 2.68 | 7.22 | 5.59 | NA | 15.69 |
| 60 | 2445699 | 1 | 38 | DN | - | - | - | 2 | - | - | 104 | 518.12 | 11.207 | 2.1 | 4.9 | 8.99 | NA | 130.64 |
| 61 | 2446615 | 2 | 50 | DN | - | - | - | 2 | - | - | 80 | 611.7 | 6.132 | 1.85 | 5.71 | 4.97 | NA | 70.10 |
| 62 | 2436276 | 2 | 43 | DN | - | - | - | 2 | - | - | 44 | 434.69 | 9.873 | 2.11 | 2.67 | 4.13 | NA | 226.67 |
| 63 | 2442768 | 2 | 31 | MCD | - | - | - | 0 | - | - | 127 | 63.9 | 111.41 | 1.2 | 3.62 | 0.89 | NA | 7.11 |
| 64 | 2444080 | 2 | 24 | MCD | - | - | - | 0 | - | - | 106 | 54 | 127.235 | 0.99 | 7 | 5.32 | NA | 1.82 |
| 65 | 2445447 | 1 | 37 | MCD | - | - | - | 0 | - | - | 195 | 105.83 | 77.007 | 2.42 | 12.16 | 5.11 | NA | 6.63 |
| 66 | 2446762 | 2 | 43 | MCD | - | - | - | 0 | - | - | 114 | 178.2 | 29.018 | 5.14 | 9.26 | 22.77 | NA | 90.75 |
| 67 | 2442337 | 1 | 43 | MCD | - | - | - | 0 | - | - | 152 | 134.3 | 54.198 | 3.22 | 12.96 | 16.99 | NA | 23.73 |
| 68 | 2443770 | 1 | 31 | MCD | - | - | - | 0 | - | - | 155 | 69.69 | 119.754 | 1.5 | 4 | 14.47 | NA | 36.17 |
| 69 | 2448575 | 2 | 38 | MCD | - | - | - | 0 | - | - | 116 | 72 | 92.46 | 0.7 | 4.87 | 3.21 | NA | 6.72 |
| 70 | 2442410 | 2 | 30 | MN | - | - | - | 0 | - | - | 112 | 67.7 | 104.627 | 1.47 | 6.01 | 2.57 | NA | 108.67 |
| 71 | 2442709 | 2 | 38 | MN | - | - | - | 0 | - | - | 145 | 48.64 | 116.864 | 3.65 | 7.22 | 3.72 | NA | 3.19 |
| 72 | 2442712 | 2 | 30 | MN | - | - | - | 0 | - | - | 133 | 60.9 | 117.252 | 3.8 | 18.76 | 3.68 | NA | 61.76 |
| 73 | 2444216 | 2 | 30 | MN | - | - | - | 0 | - | - | 139 | 57.4 | 119.558 | 1.87 | 11.96 | 1.82 | NA | 8.51 |
| 74 | 2444092 | 1 | 58 | MN | - | - | - | 0 | - | - | 169 | 96.08 | 73.124 | 7.78 | 4.59 | 2.89 | NA | 10.90 |
| 75 | 2448625 | 1 | 46 | MN | - | - | - | 0 | - | - | 140 | 54.77 | 116.514 | 1.69 | 3.59 | 1.82 | NA | 43.45 |
| 76 | 2442339 | 2 | 31 | MN | - | - | - | 0 | - | - | 125 | 51.9 | 122.721 | 2.2 | 6.67 | 1.62 | NA | 71.04 |
| 77 | 2443098 | 1 | 38 | MN | - | - | - | 0 | - | - | 146 | 78 | 106.579 | 4.39 | 8.34 | 11.5 | NA | 74.11 |
| 78 | 2444169 | 1 | 40 | MN | - | - | - | 0 | - | - | 159 | 79.5 | 88.095 | 3.45 | 7.32 | 4.58 | NA | 12.96 |
| 79 | 2446616 | 1 | 47 | MN | - | - | - | 0 | - | - | 141 | 78.9 | 99.579 | 2.51 | 5.94 | 2.72 | NA | 21.19 |
| 80 | 2446499 | 1 | 39 | MN | - | - | - | 0 | - | - | 147 | 88.9 | 91.792 | 1.84 | 9.04 | 5.72 | NA | 16.95 |
| 81 | 2447214 | 2 | 55 | MN | - | - | - | 0 | - | - | 146 | 51 | 102.04 | 2.7 | 5.79 | 2.3 | NA | 35.73 |
| 82 | 2446653 | 1 | 39 | MN | - | - | - | 0 | - | - | 148 | 124.7 | 60.971 | 2.94 | 8.69 | 15.76 | NA | 38.93 |
| 83 | 2447526 | 1 | 49 | MN | - | - | - | 0 | - | - | 145 | 85.09 | 90.219 | 4.24 | 5 | 8.59 | NA | 23.25 |
| 84 | 2448662 | 1 | 55 | MN | - | - | - | 0 | - | - | 159 | 88 | 83.0496 | 2.11 | 4.72 | 3.18 | NA | 8.96 |
| 85 | 2448811 | 2 | 40 | MN | - | - | - | 0 | - | - | 122 | 66.94 | 96.808 | 1.3 | 7.72 | 1.96 | NA | 71.78 |
| 86 | 2448406 | 2 | 40 | MN | - | - | - | 0 | - | - | 92 | 144.1 | 30.815 | 1.13 | 4.43 | 1.48 | NA | 10.53 |
| 87 | 2442351 | 2 | 45 | MN | - | - | - | 0 | - | - | 117 | 79.1 | 76.386 | 3.15 | 13.13 | 13.67 | NA | 29.32 |
| 88 | 2446681 | 1 | 32 | MN | - | - | - | 0 | - | - | 149 | 79.1 | 112.883 | 2.23 | 5.41 | 0.24 | NA | 1.07 |
| 89 | 2442970 | 1 | 24 | MN | - | - | - | 1 | - | - | 134 | 116.3 | 75.276 | 3.28 | 4.88 | 2.59 | NA | 16.56 |
| 90 | 2445530 | 2 | 29 | MN | - | - | - | 1 | - | - | 82 | 1111.3 | 3.577 | 2.48 | 5.99 | 17.14 | NA | 208.01 |
| 91 | 2446932 | 2 | 57 | MN | - | - | - | 0 | - | - | 133 | 54.1 | 98.745 | 1.59 | 5.53 | 7.57 | NA | 101.93 |
| 92 | 2443551 | 2 | 57 | MN | - | - | - | 0 | - | - | 133 | 56.28 | 97.47 | 3.95 | 4.6 | 6.71 | NA | 17.72 |
| 93 | 22HCM1 | 1 | 50 | Healthy control | - | - | - | - | - | - | 171 | 67.03 | 109.58 | 1.04 | 4.45 | 0.00 | 0.92 | 0.10 |
| 94 | 22HCM2 | 1 | 50 | Healthy control | - | - | - | - | - | - | 155 | 68.5 | 108.87 | 0.69 | 4.07 | 0.00 | 1.26 | 0.04 |
| 95 | 22HCM3 | 1 | 38 | Healthy control | - | - | - | - | - | - | 151 | 71.62 | 115.74 | 0.95 | 4.8 | 0.00 | 0.35 | 0.79 |
| 96 | 22HCM4 | 1 | 34 | Healthy control | - | - | - | - | - | - | 156 | 73.7 | 117.63 | 1.85 | 5.37 | 0.00 | 0.15 | 0.52 |
| 97 | 22HCM5 | 1 | 52 | Healthy control | - | - | - | - | - | - | 118 | 74.5 | 104.83 | 1.31 | 6.61 | 0.00 | 0.42 | 0.27 |
| 98 | 22HCF1 | 2 | 50 | Healthy control | - | - | - | - | - | - | 123 | 78.9 | 106.38 | 1.08 | 6.56 | 0.00 | 0.63 | 2.09 |
| 99 | 22HCF2 | 2 | 48 | Healthy control | - | - | - | - | - | - | 156 | 79.94 | 106.04 | 1.41 | 3.56 | 0.00 | 0.95 | 0.58 |
| 100 | 22HCF3 | 2 | 53 | Healthy control | - | - | - | - | - | - | 131 | 83.8 | 97.13 | 0.98 | 6.25 | 0.00 | 1.44 | 0.21 |
| 101 | 22HCF4 | 2 | 36 | Healthy control | - | - | - | - | - | - | 156 | 84.45 | 106.97 | 0.79 | 3.6 | 0.00 | 0.52 | 5.76 |
| 102 | 22HCF5 | 2 | 49 | Healthy control | - | - | - | - | - | - | 126 | 85 | 97.90 | 1.2 | 4.39 | 0.00 | 0.57 | 3.70 |
| 103 | UCTF2001 | 1 | 24 | Healthy control | - | - | - | - | - | - | 131 | 58.5 | 122.82 | 2.32 | 4.85 | 0.00 | 0.39 | 0.55 |
| 104 | UCTF2002 | 1 | 40 | Healthy control | - | - | - | - | - | - | 155 | 79.31 | 110.84 | 5.86 | 6.51 | 0.00 | 0.59 | 0.16 |
| 105 | UCTF2003 | 1 | 24 | Healthy control | - | - | - | - | - | - | 171 | 72.8 | 125.64 | 0.84 | 5.03 | 0.00 | 0.24 | 3.27 |
| 106 | UCTF2004 | 1 | 33 | Healthy control | - | - | - | - | - | - | 130 | 79.39 | 115.74 | 2.31 | 5.21 | 0.00 | 0.91 | 6.01 |
| 107 | UCTF2005 | 1 | 27 | Healthy control | - | - | - | - | - | - | 128 | 55.8 | 127.14 | 1.74 | 4.52 | 0.00 | 1.20 | 1.10 |
| 108 | UCTM2006 | 2 | 33 | Healthy control | - | - | - | - | - | - | 129 | 59.73 | 116.77 | 4.54 | 4.07 | 0.00 | 4.36 | 16.76 |
| 109 | UCTM2007 | 2 | 24 | Healthy control | - | - | - | - | - | - | 131 | 85.97 | 112.82 | 2.41 | 4.46 | 0.00 | 0.78 | 0.94 |
| 110 | UCTM2008 | 2 | 26 | Healthy control | - | - | - | - | - | - | 130 | 76.8 | 127.57 | 0.71 | 3.39 | 0.00 | 1.05 | 0.62 |
| 111 | UCTM2009 | 2 | 28 | Healthy control | - | - | - | - | - | - | 128 | 65.9 | 127.79 | 2.37 | 4.31 | 0.00 | 0.70 | 3.73 |
| 112 | UCTM2010 | 2 | 30 | Healthy control | - | - | - | - | - | - | 129 | 69.5 | 140.28 | 2.15 | 4.11 | 0.00 | 1.46 | 6.56 |
|  |  |  |  |  |  |  |  |  |  |  |  |  |  |  |  |  |  |  |
|  |  |  |  |  |  |  |  |  |  |  |  |  |  |  |  |  |  |  |
| IgAN_median | |  | 34 |  |  |  |  |  |  |  | 124 | 113 | 56.9 | 1.71 | 4.88 | 1.83 | 65.44 | 32.38 |
| IQR_1 |  |  | 29 |  |  |  |  |  |  |  | 115 | 86 | 30.6 | 1.07 | 3.75 | 0.80 | 46.69 | 11.26 |
| IQR_3 |  |  | 42 |  |  |  |  |  |  |  | 140 | 196 | 93.3 | 2.76 | 6.02 | 2.94 | 125.10 | 74.76 |
|  |  |  |  |  |  |  |  |  |  |  |  |  |  |  |  |  |  |  |
| DC_Median | |  | 40 |  |  |  |  |  |  |  | 133.00 | 87 | 85.6 | 2.11 | 5.73 | 3.70 | NA | 23.90 |
| IQR_1 |  |  | 31 |  |  |  |  |  |  |  | 106.75 | 65 | 33.6 | 1.48 | 4.60 | 2.36 | NA | 11.41 |
| IQR_3 |  |  | 49 |  |  |  |  |  |  |  | 146.00 | 142 | 106.1 | 3.10 | 7.62 | 6.96 | NA | 71.59 |
|  |  |  |  |  |  |  |  |  |  |  |  |  |  |  |  |  |  |  |
| HC_Median | |  | 35 |  |  |  |  |  |  |  | 131.00 | 74 | 114.3 | 1.36 | 4.49 | 0.00 | 0.74 | 0.86 |
| IQR_1 |  |  | 28 |  |  |  |  |  |  |  | 128.75 | 68 | 106.8 | 0.97 | 4.10 | 0.00 | 0.50 | 0.46 |
| IQR_3 |  |  | 49 |  |  |  |  |  |  |  | 155.25 | 80 | 123.5 | 2.31 | 5.25 | 0.00 | 1.09 | 3.70 |
