# Supplementary material for: Discovery of Fibrinogen γ-chain as a potential urinary biomarker for renal interstitial fibrosis in IgA nephropathy
Source: BMC Nephrol. 2023 Mar 20;24:60. doi: 10.1186/s12882-023-03103-7 (PMC10029243; doi:10.1186/s12882-023-03103-7)
Supplement: Supplementary file 5 — Additional file 5 [file 12882_2023_3103_MOESM5_ESM.docx]

Supplementary file legend

Additional file 5. FGG expression in microarray (GSE104954)

| No. | Group | FGG_expression |
| --- | --- | --- |
| GSM2810941 | IgAN | 3.391233 |
| GSM2810942 | IgAN | 3.859076 |
| GSM2810943 | IgAN | 3.748945 |
| GSM2810944 | IgAN | 4.878416 |
| GSM2810945 | IgAN | 3.958487 |
| GSM2810946 | IgAN | 4.077051 |
| GSM2810947 | IgAN | 4.027246 |
| GSM2810948 | IgAN | 8.753624 |
| GSM2810949 | IgAN | 3.917237 |
| GSM2810950 | IgAN | 4.014077 |
| GSM2810951 | IgAN | 4.055387 |
| GSM2810952 | IgAN | 4.600284 |
| GSM2810953 | IgAN | 3.832792 |
| GSM2810954 | IgAN | 4.604589 |
| GSM2810955 | IgAN | 4.309942 |
| GSM2810956 | IgAN | 4.316692 |
| GSM2810957 | IgAN | 4.030073 |
| GSM2810958 | IgAN | 4.820911 |
| GSM2810959 | IgAN | 7.745577 |
| GSM2810960 | IgAN | 3.947594 |
| GSM2810961 | IgAN | 3.745776 |
| GSM2810962 | IgAN | 3.836356 |
| GSM2810963 | IgAN | 3.872787 |
| GSM2810964 | IgAN | 4.775689 |
| GSM2810965 | IgAN | 4.619208 |
| GSM2811043 | living kindy donor | 3.840582 |
| GSM2811044 | living kindy donor | 3.586374 |
| GSM2811045 | living kindy donor | 3.688692 |
| GSM2811046 | living kindy donor | 3.561235 |
| GSM2811047 | living kindy donor | 3.833004 |
| GSM2811048 | living kindy donor | 3.726601 |
| GSM2811049 | living kindy donor | 3.686225 |
| GSM2811050 | living kindy donor | 3.452472 |
| GSM2811051 | living kindy donor | 3.655987 |
| GSM2811052 | living kindy donor | 3.877256 |
| GSM2811053 | living kindy donor | 3.533103 |
| GSM2811054 | living kindy donor | 3.528222 |
| GSM2811055 | living kindy donor | 3.629093 |
| GSM2811056 | living kindy donor | 3.502759 |
| GSM2811057 | living kindy donor | 3.751403 |
| GSM2811058 | living kindy donor | 3.636234 |
| GSM2811059 | living kindy donor | 3.775488 |
| GSM2811060 | living kindy donor | 4.243947 |
| GSM2811026 | living kindy donor | 3.954414 |
| GSM2811027 | living kindy donor | 4.114619 |
| GSM2811028 | living kindy donor | 3.983498 |
